# How can we discover developable antibody-based biotherapeutics?

**DOI:** 10.3389/fmolb.2023.1221626

**Published:** 2023-08-07

**Authors:** Joschka Bauer, Nandhini Rajagopal, Priyanka Gupta, Pankaj Gupta, Andrew E. Nixon, Sandeep Kumar

**Affiliations:** ^1^ Early Stage Pharmaceutical Development Biologicals, Boehringer Ingelheim Pharma GmbH & Co. KG, Biberach/Riss, Germany; ^2^ In Silico Team, Boehringer Ingelheim, Hannover, Germany; ^3^ Biotherapeutics Discovery, Boehringer Ingelheim Pharmaceuticals Inc., Ridgefield, CT, United States

**Keywords:** biotherapeutics, drug discovery and development, developability, biopharmaceutical informatics, machine learning, computational biophysics

## Abstract

Antibody-based biotherapeutics have emerged as a successful class of pharmaceuticals despite significant challenges and risks to their discovery and development. This review discusses the most frequently encountered hurdles in the research and development (R&D) of antibody-based biotherapeutics and proposes a conceptual framework called biopharmaceutical informatics. Our vision advocates for the syncretic use of computation and experimentation at every stage of biologic drug discovery, considering developability (manufacturability, safety, efficacy, and pharmacology) of potential drug candidates from the earliest stages of the drug discovery phase. The computational advances in recent years allow for more precise formulation of disease concepts, rapid identification, and validation of targets suitable for therapeutic intervention and discovery of potential biotherapeutics that can agonize or antagonize them. Furthermore, computational methods for *de novo* and epitope-specific antibody design are increasingly being developed, opening novel computationally driven opportunities for biologic drug discovery. Here, we review the opportunities and limitations of emerging computational approaches for optimizing antigens to generate robust immune responses, *in silico* generation of antibody sequences, discovery of potential antibody binders through virtual screening, assessment of hits, identification of lead drug candidates and their affinity maturation, and optimization for developability. The adoption of biopharmaceutical informatics across all aspects of drug discovery and development cycles should help bring affordable and effective biotherapeutics to patients more quickly.

## 1 Introduction

Since the inception of hybridoma technology, which facilitated large-scale monoclonal antibody (mAb) production, biotherapeutics have experienced significant growth (Koehler and Milstein, 1975). The Food and Drug Administration’s (FDA) approval of the pioneering mAb therapeutic, muromonab or Orthoclone OKT3, in 1986 (Smith, 1996), set the stage for numerous groundbreaking developments in biotherapeutics. As of 2022, over 110 approved mAbs and more than 65 mAbs in phase-2/3 and phase-3 clinical trials have emerged (Kaplon et al., 2022). Clinically, mAbs have demonstrated their efficacy in treating serious conditions such as neurodegenerative diseases, autoimmune diseases, and diverse types of cancers (Reichert et al., 2009; Lu et al., 2020).

Despite the promising trajectory of biotherapeutics, the biopharmaceutical industry faces mounting pressure due to decreasing productivity and increasing research and development (R&D) costs. The average R&D cost surged from $1.2 billion in 2007 (adjusted United States dollar value of $1.6 billion in 2020) to $2.8 billion in 2016 (equivalent to $3.1 billion in 2020) (DiMasi and Grabowski, 2007; DiMasi et al., 2016; Farid et al., 2020). Concurrently, the success rate of phase-1 to approval dropped from 30% in 2007 to 12% or lower in 2016 (Farid et al., 2020). These trends suggest the presence of several challenges along various stages of discovery and development of novel biological therapeutics. A lack of detailed understanding of disease biology, the inability of model systems to reliably predict human diseases and outcomes of therapeutic interventions, the lack of efficacy, target-mediated toxicity and other safety issues, and suboptimal developability profiles are among the major reasons that may contribute to drug failures during clinical trials (Mehta et al., 2017; Fogel, 2018). The identification of new targets presents additional challenges toward development of novel therapeutic concepts and discovery of multi-specific biotherapeutics, resulting in low approval rates despite high development costs (Swinney and Anthony, 2011). Next-generation biotherapeutics such as nanobodies, bi- and multi-specific antibodies, and T-cell receptor mimetics are broadening clinical applications (Strohl, 2018); however, these novel formats are often more challenging to develop into marketed biologic drug products (Runcie et al., 2018; Wang et al., 2019; Sawant et al., 2020). Furthermore, as the biopharmaceutical industry shifts its focus toward patient convenience, drug product development processes must be tailored to emerging routes of drug administration such as subcutaneous or intravitreal delivery, necessitating high-concentration protein formulations (HCPFs) (Garidel et al., 2017). These requirements introduce additional challenges to the manufacturability and developability of novel drugs. Integrating developability early in the drug discovery process can help avoid costly delays or failures at later stages and potentially increase the likelihood of success during clinical trials and approvals. Numerous technological advancements have been made since the approval of the first mAb to overcome challenges in the R&D pipelines and accelerate novel drug discovery and development (Martin et al., 2023). However, every new technology comes with associated risks and limitations (Gray A. C. et al., 2020; González-Fernández et al., 2020).

*In silico* techniques have been well established in small-molecule drug discovery (Shaker et al., 2021). Over the past decade, considerable progress has been made toward developing *in silico* strategies for the discovery and development of biologic drugs as well. In fact, developability has emerged as a key concept for biologic drugs over this time (Jarasch et al., 2015; Kumar and Singh, 2015; Bailly et al., 2020; Garripelli et al., 2020; Khetan et al., 2022; Mieczkowski et al., 2023). A variety of computational tools and procedures are now employed across various stages of drug development, such as hit selection, lead identification, optimization, affinity maturation, and early developability assessment. However, a significant potential of *in silico* technologies toward the discovery of biotherapeutics still remains untapped. As collaborative academic and industrial initiatives continue to demonstrate the viability of *in silico* antibody discovery techniques, it is important to acknowledge that the nascent nature of these methods often results in a lack of historical evidence to support their success and therefore requires a cultural shift toward proactive adoption of innovation to continually improve drug discovery and development processes. To address these challenges and enhance the success rate of novel targets, there is an urgent requirement for an integrated vision to create a platform that streamlines biotherapeutic discovery and development via syncretic use of experimentation and computation. Such a vision would not only accelerate the development of new biotherapeutics and reduce costs but also expand the druggable target space.

## 2 Biopharmaceutical informatics: integrating drug discovery and development

**FIGURE 1:**
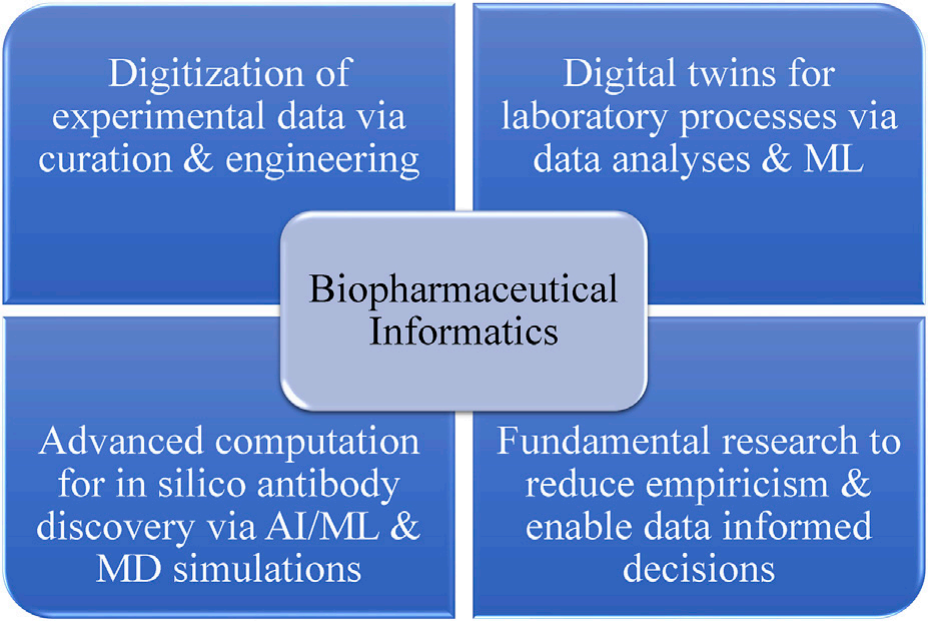
Strategic components for the vision of biopharmaceutical informatics. The digital transformation of the biopharmaceutical industry, achieved through capturing and curing experimental data, can enable the development and continuous improvement of digital twins for laboratory processes and prediction of experimental results before their execution. Fundamental research connecting molecular sequences, structures, and dynamics of biologic drug candidates can enhance our understanding of experimental observations, reduce empiricism, and enable more data-informed decision-making at various project stages. Moreover, the integration of computational learning technologies with principles of molecular modeling and simulations can potentially facilitate the *in silico* discovery of biotherapeutics. It is important to note that the key to biopharmaceutical informatics lies in the syncretic use of experimentation and computation, with a shared goal of making the discovery and development of biotherapeutics more efficient.

In the realm of biotherapeutics, it is crucial for drug candidates to be both developable and functional. Biotherapeutic drug candidates often encounter developability challenges related to manufacturing, safety, immunogenicity, efficacy, pharmacology, and drug product heterogeneity. Many of these risks can be linked to the inherent physicochemical properties of a biologic drug candidate, as determined by its protein sequence, three-dimensional structure, and molecular dynamics (MD) (Xu et al., 2018). Considering the intrinsic physicochemical properties of a biotherapeutic drug candidate, which are encoded in its amino acid sequence and structure, early in the discovery and development can help identify and mitigate risks associated with various developability issues, such as chemical, conformational, colloidal, and physical instabilities. Moreover, by employing the innovative approach of biopharmaceutical informatics, these sequence–structural attributes can be modified for improved developability as described previously by Kumar et al. (2018a). Figure 1 outlines the primary components of biopharmaceutical informatics. This interdisciplinary field advocates for the digital transformation of the biopharmaceutical industry by converting experimental data collected during drug discovery and development phases into FAIR (findable, accessible, interoperable, and reusable) information systems. These systems can be leveraged by data scientists to create predictive tools such as digital twins of actual laboratory processes. Additionally, the field promotes the increased use of AI/ML (artificial intelligence/machine learning) and computational biophysics to address fundamental challenges in drug discovery and development through research. Biopharmaceutical informatics seeks to enable data-driven decision-making at every stage of biologic drug discovery and development. Developability is a key aspect of biopharmaceutical informatics, encompassing both *in silico* tools and experimental studies such as developability assessments. Rooted in the energy landscape theory, the concept of developability posits that the conformational ensembles and potential energy landscapes of large macromolecules, like mAbs, change with their environment (e.g., pH, temperature, and physicochemical state) (Onuchic, 1997; Ma et al., 2000; Kumar et al., 2009). As a result, the physicochemical properties of conformational ensembles of biotherapeutics under a given set of environmental conditions dictate their biophysical experiment outcomes. If proteins with the same size and fold are analyzed under identical conditions using standardized experiments, differences in the results should be attributable to sequence–structural variations among the proteins. The ability to predict experimental outcomes by analyzing the sequence–structural characteristics of biotherapeutic drug candidates is a primary goal for biopharmaceutical informatics, as part of the development of computational methods that facilitate discovery of antibodies *in silico* (DAbI).

Optimal synergies and benefits can be achieved by integrating cost-effective, rapid computational methods with standardized biophysical experimental studies, which are characteristic of current developability assessments in biologic drug discovery and early-stage product development (Zurdo, 2013; Jarasch et al., 2015; Xu et al., 2018). Late-stage development approaches typically focus on assessing the changing conditions of a single molecule in the drug manufacturing process using quantitative unit operation models (Smiatek et al., 2020), while early-stage approaches require analyzing a diverse set of molecules under identical conditions. Biopharmaceutical informatics plays a pivotal role in bridging the gap between biologic drug discovery and development by improving the understanding of the relationship between macromolecular sequence–structure–function and developability.

A key challenge in biopharmaceutical informatics is correlating the “macroscopic” experimentally determined properties of a biologic with its “microscopic” sequence–structure features computed *in silico*. Uncovering these correlations can guide molecular sequence optimization strategies, proactively addressing potential obstacles in drug product development by predicting the performance of the final drug candidate in the streamlined platform processes used during development stages. This process necessitates combining data from standardized biophysical experiments with descriptors computed from molecular modeling and simulations in a common database. Various statistical and machine learning approaches can be employed to develop mathematical models that predict the solution behavior of mAbs based solely on their sequence–structure information, depending on the available data (Tomar et al., 2016; Jain et al., 2017b; Chiu et al., 2019; Hebditch and Warwicker, 2019; Lecerf et al., 2019; Raybould et al., 2019; Starr and Tessier, 2019; Kuroda and Tsumoto, 2020; Zhang et al., 2020). As a result, the interdisciplinary field of biopharmaceutical informatics aims to seamlessly integrate techniques from computational and experimental biophysics, information technology, and data science to provide data-driven inputs for the decision-making framework for all stages of biologic drug discovery and development.

## 3 Opportunities for computation at various stages of biotherapeutic discovery and early development

There are numerous opportunities to collaboratively apply computational and experimental tools to facilitate faster and more efficient drug engineering and development. In this review article, we present a diverse set of use cases at various stages of biotherapeutics discovery and development projects that could benefit with increased use of computation in synchrony with the experiments to demonstrate the practical feasibility of our vision. The major challenges faced at distinct stages of biotherapeutic discovery and early drug development are described in Table 1 along with potential computational opportunities to address them. The pros and cons of these computational opportunities are also presented in Table 1. It is important to note that the field has not matured uniformly across all stages of discovery and development cycles for biotherapeutics. For example, computational approaches to developability assessments and lead optimization (LO) are currently more advanced than *in silico* antibody discovery and *in silico* formulation development. Moreover, there are also opportunities to modify the workflows and transitions between the different discoveries and development stages in view of the rapidly growing capabilities of computation. These opportunities are described in the following sections.

**TABLE 1:** Opportunities for the expanded use of computational approaches throughout the discovery and development process of biotherapeutics.

Process stage	Typical problems	Potential applications of computational approaches	Pros	Cons
*In vitro* synthesis of immunogens/antigens to generate corresponding antibodies	1. Availability of structural models for immunogens and accurate definition of epitope(s) of therapeutic interest 2. Aggregation tendency, protein insolubility, and reduced conformational stability may result in limited material availability for immunization experiments 3. Epitope(s) of therapeutic interest might not be immunodominant	1. Protein structure prediction and precise definition of epitope(s) of therapeutic interest 2. Sequence/structure-based optimization for improved solubility via APR disruption, supercharging; and increased conformational stability via residue scan can help improve quantity as well as quality of material needed for immunization 3. Strategies for disruption or masking of immunodominant but therapeutically irrelevant epitopes to improve chance of antibody binders to the therapeutically relevant epitopes	1. Protein structure is crucial for structure-based approaches to drug discovery defining epitopes of therapeutic interest. The emergence of AI-based protein structure prediction methods has enhanced the structural definition of immunogens in recent years 2. Judiciously selected mutations at single or multiple sites can significantly improve the availability of immunogen material in the laboratory	1. Confidence levels in different regions of the structure should be considered, as flexible regions are typically predicted with lower confidence levels 2. Defining the epitope(s) of therapeutic interest and avoiding mutations in and around them is important 3. Implementing site-directed mutagenesis of immunogens to improve material availability also requires a cultural shift among experimental scientists
Antibody generation	1. Animal immunizations can be time-consuming, expensive, and may yield inconsistent results 2. The lead antibody molecule identified through animal immunization may necessitate humanization and developability enhancements 3. Humanized mice and display technologies do not entirely capture the complete human immunome 4. Phage and yeast display technologies can quickly identify high-affinity binders, but these may require further optimization for developability	1. Generative AI can aid in designing antigen-specific and agnostic libraries with incorporated developability features 2. Virtual screening of antibody libraries against given antigen(s)/epitope(s), followed by docking and structure-based affinity enhancements 3. Utilizing computational methods to design phage and yeast display libraries for enhanced developability and/or affinity 4. Employing computational approaches to redesign antibody CDRs for altered specificities	1. Adopting computational methods can reduce timelines and costs associated with antibody discovery 2. Expanded druggable antigen space 3. Opportunities to explore a broader sequence diversity, thereby maximizing the odds for antibody discovery compared to conventional methods 4. Addressing developability during library design can help reduce time required for lead optimization	1. Emerging technology 2. Necessitates more extensive validation and experimental demonstration of its capabilities before routine project use 3. Requires a cultural shift from experimentally driven antibody discovery to computationally driven approaches
Hit selection and lead identification	1. Sequencing of identified hits 2. Epitope mapping of the hits to ensure the desired therapeutic effect in the absence of structural models for the antigen-antibody complex 3. Experimental evaluation of several hundreds of candidates for functionality and developability can be time and resource-intensive	1. Establishment of suitable sequencing pipelines 2. Computational prediction of epitopes and paratopes for epitope mapping purposes 3. In-silico evaluations of candidates for developability and manufacturability can facilitate the selection of developable hits and identification of lead candidate(s) with favorable developability characteristics 4. Development of digital twins for biophysical processes via computational biophysics and data science	1. Incorporation of computational assessments can aid in guiding hit selection for experimental testing 2. Proactive consideration of developability can help reduce costs and efforts to identify lead molecules 3. Opportunities to enhance our understanding of the connection between molecular sequence-structural properties and experimental outcomes	1. Greater availability of data is needed to connect 'microscopic' sequence-structural features of antibodies with the 'macroscopic' biophysical outcomes 2. Lack of digitization and digital transformation present significant challenges 3. A cultural shift from protecting experimental data to sharing it with computational scientists is required among discovery scientists
Lead optimization	Lead candidates may require humanization, affinity optimization, and elimination of physicochemical liabilities in the CDRs for enhanced developability	1. Structure-based modeling of the lead candidates can assist in their humanization, affinity maturation, and identification of potential sequence/structural motifs that may contribute to their physicochemical degradation. Access to this information can help direct protein engineering strategies for lead optimization 2. Assessment of the optimized lead candidates for their drug likeness	1. Computational guidance for lead optimization efforts can decrease timelines and costs 2. This aspect represents the most developed application of computational protein design in biotherapeutic drug discovery 3. Numerous well-developed computational solutions are available	1. There remains cultural resistance to the adoption of computational protein design for lead optimization among industrial scientists 2. Greater dissemination of successful case studies, where computational protein design makes a difference, is needed to raise awareness
Early stage developability assessments	1. Assessing molecular stability and compatibility of drug candidates, identified during drug discovery, with platform processes utilized in drug development 2. Adapting to multiple product development goals such routes of administration and product presentations	1. Structure prediction of full length antibodies and novel formats 2. In-silico development of formulations 3. Employing multi-scale simulations to anticipate platform compatibility and evaluate molecular responses to stresses encountered during manufacturing, storage, and transportation 4. Utilization of predictive algorithms to determine suitable bioprocess conditions 5. Establishing digital twins for various facets of drug development	1. Developing full-length models of the drug substance can facilitate improved prediction of molecular origins of dominant degradation routes during manufacturing, storage, and shipping 2. Accelerating formulation process development and saving costs of drug development can be achieved through pH and buffer screening of antibody formulations via in-silico characterization of molecular integrity of the drug substance 3. Resource savings can be realized with the development of digital twins	1. Computationally intensive calculations 2. Need for improved correlations between experimental results and molecular simulations 3. Consistent availability of development data across different projects 4. Requirement for greater investments in the digitalization of drug development data

### 3.1 Antigen optimization

The discovery of antibody-based biotherapeutics adheres to a stepwise approach once a target antigen or multiple antigens for simultaneous targeting in a multi-specific format have been identified. The initial phase entails producing enough target antigens to enable animal immunization, *in vitro* selection of antigen-specific antibodies, and functional activity characterization. However, some antigens exhibit favorable expression *in vivo* but encounter conformational stability and solubility issues *in vitro*, outside the cellular context (Qing et al., 2022). Producing recombinant antigens can be particularly challenging for certain target classes, such as membrane proteins (G protein–coupled receptors and ion channels) (Bill et al., 2011). If antigen binding is impacted by the *in vitro* conformational stability and/or solubility of the antigen, then these issues may hinder the entire antibody discovery strategy and functional validation of the antibody hits.

Computational methods can aid in the redesign of antigens with enhanced conformational stability and solubility when a threedimensional crystal structure or model is available. Bioinformatic tools can enable crystal structure refinement, modeling of breaks and gaps, loop modeling, energy minimization and molecular dynamics simulations to support antigen redesign. When the crystal structure of an antigen is unavailable, protein structure prediction techniques can often estimate it (Nimrod et al., 2018). For example, homology-based structure modeling can be employed using crystal homologs. A sequence identity of at least 30% between the protein of interest and its crystal homologue is typically sufficient for structure generation through homology modeling. However, some novel targets may not have homologs with existing crystal structures. This can be due to the inherent difficulty in obtaining crystal structures of membrane-associated proteins, which often have poor solubility. Membrane proteins represent a significant class of drug targets, and the discovery pipeline frequently proceeds without knowledge of the antigen structure. In such challenging cases, recent groundbreaking advances in *de novo* protein structure prediction techniques have achieved remarkable success and accuracy by leveraging machine learning and deep learning algorithms (AlQuraishi, 2019; Gao et al., 2020; Pereira et al., 2021; Jones and Thornton, 2022). Deep learning–based structure prediction methods, such as AlphaFold2 and RoseTTAFold, combined with physical modeling, have outperformed numerous conventional approaches (Baek et al., 2021; Jumper et al., 2021; Pereira et al., 2021; Jones and Thornton, 2022). Understanding of the antigen’s three-dimensional structure can be crucial for accurately assessing its stability and solubility, computationally. This knowledge can also help enhance solubility without sacrificing stability and functional activity, allowing for the extraction of crystal structures, and facilitating experimental assays that measure target binding. Care should be taken, however, to minimize the impact of such mutations on the overall molecular structure of the target antigen and preserve its potential to generate adequate immune response to epitopes of therapeutic interest. Bioinformatics can also support rational strategies to immunize only therapeutically relevant epitopes on the antigen surface. This means epitopes that may be immune-dominant but are of no therapeutic interest or relevance can be either eliminated or masked to facilitate the immunization of the desired epitopes of therapeutic importance.

### 3.2 Antibody generation

Immunization strategies have long been employed to generate high-affinity antibodies, using previously expressed and purified antigens to establish immune reactions in animals (typically laboratory mice, humanized/transgenic mice, or other animals like chickens, rabbits, or cows). Antibody binding to specific antigens can be obtained through techniques such as hybridoma (Koehler and Milstein, 1975), single B cells (Yu et al., 2008), or screening natural and/or synthetic antibody libraries via display technologies using phage or yeast (Benatuil et al., 2010; Chen and Sidhu, 2014; Alfaleh et al., 2020; Gray A. et al., 2020; Nagano and Tsutsumi, 2021; Ledsgaard et al., 2022; Valldorf et al., 2022). Promising candidates are selected and validated using antigen-binding assays that align with the research target profile. Currently used methods in the biopharmaceutical industry for antibody generation are almost exclusively experimental, and depending on the techniques used, it can take several months before an initial set of antibody-based binders is available for further investigation and lead identification. Fully synthetic human antibody libraries containing Fabs chosen for their biophysically favorable development characteristics have been developed using experimental means (Valldorf et al., 2022). Special emphasis has been placed on selecting molecules with enhanced chemical, conformational, and colloidal stabilities (Tiller et al., 2013). The availability of such libraries can significantly help accelerate the discovery of antibody-based biotherapeutics by pre-paying for developability.

The concept of optimized antibody libraries for generating developable antibodies can be integrated with *de novo* computational databases containing an immense variety of human-like light- and heavy-chain combinations (Pan and Kortemme, 2021; Akbar et al., 2022). Targeted mutations at specific sequence positions [e.g., complementarity-determining regions (CDRs)] in the antibody sequences could further broaden the library, either to recognize different antigens or to optimize binding affinity toward a specific antigen (Ledsgaard et al., 2022). Recently, a generative adversarial network was successfully employed to create a diverse library of novel antibodies that emulate somatically hypermutated human repertoire responses (Amimeur et al., 2020). This *in silico* method further revealed residue diversity throughout the variable region, which could be useful for additional computational tools like CDR redesign. CDR redesign utilizes a highly developable antibody framework and modifies the original CDRs, or paratope, to recognize a new antigen. In recent years, noteworthy progress has been made in designing not only thermodynamically stable but also biologically functional antibodies (Baran et al., 2017).

Computational technologies, initially developed for small-molecule drug discovery, can also be applied to antibody-based drug discovery. Once fully developed and implemented, these computational methods will provide additional means to generate diverse antibody binders against a target antigen. These methods will not only help reduce animal use in biologic drug discovery but also decrease reliance on experimental trial and error for finding initial hits. Initial case studies describing such methods are beginning to emerge in the literature (bioRxiv.org for preprints) (Sever et al., 2019; Wilman et al., 2022). Additionally, it becomes feasible to find potential binders to difficult targets, thereby expanding the druggable target space for antibody-based biotherapeutics.

**FIGURE 2:**
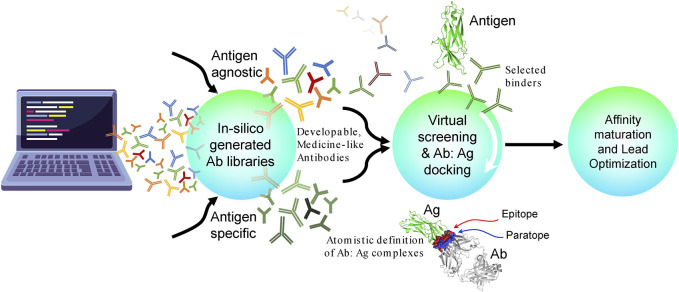
Conceptual roadmap for the discovery of antibodies *in silico* (DAbI). This conceptual roadmap can be divided into three major parts that can be developed either independently or in synchrony. The first part focuses on the *in silico* generation of medicine-like, antigen-agnostic, or specific antibody sequence libraries. Several machine learning algorithms are currently being developed to facilitate the *in silico* generation of antibodies. In the second part, these *in silico* generated antibodies and their structural models can be used to screen against a given antigen or an epitope on an antigen via virtual screening, docking, or other computational chemistry-based algorithms. Conversely, a large set of potential antigens can also be pre-screened against the antibody libraries using the same computational technologies. In both cases, the goal is to obtain atomistic definitions of putative antibody–antigen complexes. At this stage, it is preferable to virtually screen a larger number of antibodies (e.g., 1–10 million) and then select a much smaller number (e.g., 10–100) for docking simulations. This will help speed up the calculations and save computational resources. It is also important to quantitatively assess the quality of modeled antibody–antigen complexes by comparing them against crystal structures of other antigen–antibody complexes. A third option is to convert the whole or portions of the *in silico* generated antibody libraries into molecular libraries suitable for phage or yeast display and then pan them against a diverse panel of desired antigens. In the third part, the structural models of the putative antibody–antigen complexes obtained previously can be used to identify potential lead antibody candidates and modify their binding affinities to the desired levels via single- or multi-residue mutations in the paratope regions through computational protein design. These structural models can also be used to impart cross-reactivity to homologous antigens from other non-human species and/or to even create surrogate antibodies. Care should be taken to avoid introducing residues susceptible to physicochemical degradation and therefore reducing the developability of the lead candidates. It is important to note that DAbI will require changing the discovery workflows because it is pre-paying for developability and may therefore require significantly reduced effort during lead optimization (LO).

Figure 2 provides an overall conceptual roadmap for Discovery of antibodies in silico (DAbI). The proposed roadmap encompasses three major parts where each part can have multiple stages depending upon the project in hand. In the first part, the key is to use different computational algorithms to generate medicine-like human antibody sequence libraries *in silico*. These libraries can be either antigen-specific or antigen-agnostic and are of orthogonal utilities. For example, creation of antigen- or epitope-specific antibody libraries via machine learning can help us achieve early success in each antibody discovery project by facilitating a focused path to the discovery of lead candidates toward the antigen and support the therapeutic concept. A biological analog of such libraries shall be the sequence repertoires obtained from immunized animals, hybridomas, or the results obtained by panning the display libraries against a specific antigen. However, such libraries have to be generated repeatedly for each different antigen or epitope. Antigen-/epitope-agnostic libraries on the other hand can be incredibly useful toward supporting multiple drug discovery projects simultaneously. Such libraries can be thought of as naive B-cell repertoires obtained from humanized animals prior to immunization with specific antigens. The computationally generated naive antibody repertoires can potentially capture greater sequence diversities than those feasible from humanized animals, display technologies, or observable B-cell repertoires. Within a discovery organization, such libraries have to be constructed only once and be potentially useful toward pre-computation of binders for all the targets of interest to the organization. These pre-computed antibody binder libraries can potentially accelerate early antibody discovery projects because now the discovery process does not have to wait for availability of target reagent in the laboratory. Therefore, such libraries can be particularly useful toward difficult to express and purify targets such as membrane proteins. Irrespective of the purpose of *in silico* generated antibody libraries, it is important to generate structural models of (at least) the variable regions of the antibodies sampled from these libraries. The generated structures can then be used for assessing their medicine-likeness and developability. Early elimination of non–medicine-like antibodies from such libraries can improve their utility and differentiate them from those generated using the experimental means solely. The structural models can also be used for predicting antibody paratopes. Many computational methods are currently available for the structural prediction of antibodies. The major challenges in this field include prediction of HCDR3 conformation and pairing of the light- and heavy-chain variable regions (Fernández-Quintero et al., 2023).

In addition to the design of the *in silico* antibody libraries, currently available computational methods also provide an opportunity to design single or a few human antibody variable regions against specific antigen epitopes *de novo* (Chowdhury et al., 2018; Nimrod et al., 2018). The design process can also commence with a structural model of an antigen:antibody (Ag:Ab) complex, generated using molecular docking of the antigen and antibody structures (Nimrod et al., 2018). Subsequently, the affinity of the antigen toward the antibody can be either altered by randomly introducing sequence variations or selectively re-designing interfaces using structure-based approaches (Nimrod et al., 2018). For example, interfacial residues in the Ag:Ab complexes that significantly contribute to their stability and instability can be identified through computational alanine (Ala) scanning. In the following step, the identified residue positions can be scanned for mutations that either increase or decrease the stability of the Ag:Ab complex and enhance or reduce the affinity of the antibody toward its cognate antigen (Sheng et al., 2022), depending on the project requirements. Another appealing alternative for rational antibody design involves hotspot grafting with CDR loop swapping, which only requires information about interactions with the antigen (Liu et al., 2017).

The goal of epitope-driven antibody generation is to design an antibody variable region with a paratope that complements the given epitope. Since CDRs make up most of the paratope, initial efforts to design epitope-specific antibodies have focused on *ab initio* CDR redesign and modeling. OptCDR (Pantazes and Maranas, 2010), used in conjunction with Rosetta Antibody Modeler, generates epitope-specific high-affinity CDRs by selecting the most feasible canonical loop conformations followed by iterative model optimization and improvements in binding energy. This method enables the generation of a focused library of antibody binders, quite like hit sequences obtained from experiments. OptCDR was later optimized (OptMAVEn) to consider the entire fragment variable (Fv) region rather than just CDRs as the starting point for generating antibody binders (Li et al., 2014), allowing for the incorporation of humanness at the antibody generation stage through careful selection of human framework region residues. Further advances have incorporated MD simulations for accurate evaluation of binding energetics (Chowdhury et al., 2018). A one-to-one residue matching method called epitoping, which starts from antibody structures with basic shape complementarity, was developed to obtain an accurate epitope–paratope binding match (Nimrod et al., 2018). Although this process requires a pre-identified approximate match, it can be considered for lead optimization to improve binding.

Recent advancements in generative deep learning and the availability of approximately 2,000 solved crystal structures of the antibody–antigen complexes have opened possibilities for structure-based *de novo* antibody generation. A proof-of-concept study utilizing a variational autoencoder (VAE)–based generative algorithm demonstrated the capability to directly generate 3D coordinates of antibody backbones that complement a specific epitope (Eguchi et al., 2022). Additionally, another deep learning algorithm was developed to learn the 3D features of antibodies from 1D sequences, enabling the generation of antibody sequences with desired structural characteristics (Akbar et al., 2022). Although the proof-of-concept study primarily aimed to achieve high-affinity binder antibody sequences for a given epitope, the method holds potential for encoding additional features, allowing the model to be tailored to produce highly developable sequences. As stated previously, generation of epitope-specific antibodies or libraries thereof has immediate applications for individual drug discovery projects, since the knowledge of epitopes is often required for defining novel therapeutic concepts.

### 3.3 Early screening for developability of *in silico* generated antibody libraries

Once the *in silico* antibody sequence libraries have been generated, it is worth assessing the generated antibody sequences for developability and advancing highly developable sequences to further stages of discovery. The developability assessment tools to be employed here can be ported over easily from those used at the hit selection and lead identification, lead optimization, and early development stages in the conventional biotherapeutic discovery and development workflows.

Lipinski’s “rule-of-five” revolutionized the discovery and development of small molecules by providing guidelines for improving their solubility and permeability (Lipinski, 2000). However, establishing similar rules for new biological entities (NBEs) has proven challenging due to their complex structures. In response, researchers have turned to biophysical evaluations and computational approaches to better understand these entities and overcome inherent obstacles. Biophysical evaluations of clinical-stage antibodies have contributed to the empirical definition of analogous boundaries, offering valuable insights for NBE development (Jain et al., 2017b; Raybould et al., 2019; Jain et al., 2023). Additionally, marketed antibodies have been profiled using calculated physicochemical descriptors, in an approach known as the DEvelopability Navigator *In Silico* (DENIS) (Ahmed et al., 2021; Licari et al., 2022). These advances have significantly contributed to our understanding of NBEs and their development processes.

Biotherapeutics can undergo various levels of conformational changes over time, which presents significant challenges regarding conformational stability during manufacturing, shipping, and storage. This is because the environment of a biotherapeutic drug candidate can influence its structure, highlighting the importance of understanding these complex molecules in more detail. To address this, biophysical analysis employs a variety of techniques, such as thermodynamic, spectroscopic, and hydrodynamic methods, for characterizing protein-based drug candidates. These techniques are routinely used during the discovery phase to guide the identification and characterization of the lead drug candidates. Some properties commonly assessed during biophysical analysis include post-translational modifications (e.g., glycosylation, deamidation, isomerization, oxidation, and fragmentation), aggregation, self-association, hydrophobicity, molecule pI, and viscosity for high-concentration liquid formulations. While these techniques are well established, they can be time- and resource-consuming and demand expert knowledge and advanced instrumentation. This has driven researchers to seek more efficient and accessible methods for obtaining critical data. *In silico* tools can predict the intrinsic biophysical properties of drug candidates along with identifying their degradation routes, whose knowledge is important for establishing appropriate formulation strategies. These tools demonstrate significant relationships between the Fv domain sequences and physicochemical properties that define antibody developability. For example, post-translational modification sites, such as deamidation, aspartate isomerization, oxidation, and fragmentation can be identified using computational approaches (Irudayanathan et al., 2022; Vatsa, 2022). Similarly, hydrophobic interaction chromatography (HIC) retention times have been successfully correlated with sequence and structure features through diverse methods such as quantitative structure–property relationship (QSPR) modeling and machine learning (Jain et al., 2017a; Jetha et al., 2018; Karlberg et al., 2020). Although solution and colloidal state properties are challenging to predict due to multiple influencing factors, computational tools like SOLpro and PROSO II have demonstrated their ability to predict solubility upon expression with an accuracy of ∼75% (Magnan et al., 2009; Smialowski et al., 2012). The isoelectric point (pI) is a crucial physicochemical property for mAbs. It has been associated with specific developability aspects such as thermostability, viscosity, and resistance to high molecular weight species formation at low pH. Tools like MassLynx, Vector NTI, and EMBOSS (Rice et al., 2000) calculate pI based on sequence data, achieving results within a 15% range of experimentally determined values (Goyon et al., 2017). Tools that predict the pI based on protein structure can provide a more accurate result, since the underlying residue p*K*a values are calculated by considering the residual microenvironments. Viscosity is also a critical factor in the colloidal stability of biologics and is influenced by electrostatics and hydrophobicity, which are in turn determined by the Fv sequence and structure. The *in silico* tool, spatial charge map (SCM), can identify highly viscous antibodies based on the mAb structure (Agrawal et al., 2015). Biomolecule aggregation is related to sequence and structural characteristics, such as the presence of aggregation-prone regions, hydrophobicity (Münch and Bertolotti, 2010), electrostatics (Buell et al., 2013), and dipole moments (Tartaglia et al., 2004), which enable both sequence- and structure-based computational predictions. Various *in silico* tools play a significant role in guiding mAb candidate design with high colloidal stability by predicting the impact of single or multiple amino acid exchanges on aggregation propensity. Alternative tools such as TANGO, PASTA, FoldAmyloid, SALSA, and AggreRATE-Pred can detect aggregation-prone regions based on the physicochemical principles of secondary structure elements, particularly the ability to form intermolecular cross-β-structures (Fernandez-Escamilla et al., 2004; Trovato et al., 2007; Zibaee et al., 2007; Garbuzynskiy et al., 2010; Walsh et al., 2014; Rawat et al., 2019). In summary, these *in silico* tools can effectively predict various biophysical properties of biotherapeutics. Their high-throughput capabilities make them particularly attractive for biophysical assessments during various stages of the drug discovery process.

### 3.4 Hit selection and lead identification

Following the production of antigen-binding antibodies through immunized animals, hybridoma cells, or phage and yeast display techniques, the variable regions of the antibodies are sequenced, and the binders are validated in the conventional workflows adapted by the biopharmaceutical industry. The immunization methods, strength and diversity of the immune responses, and sequencing technologies used can yield numerous unique hits, particularly via B-cell cloning and repertoire sequencing. Subsequently, these diverse hits must be prioritized to identify the most promising lead candidates, necessitating extensive resources to experimentally test each hit and confirm antigen binding.

**FIGURE 3:**
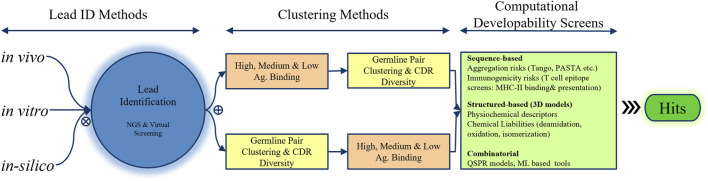
Integration of *in vivo*, *in vitro*, and *in silico* approaches for hit selection in the discovery phase of the pharmaceutical industry. Next-generation screening and virtual screening methods are employed to identify promising leads, which are then prioritized using clustering techniques based on 1) antigen binding and 2) a combination of germline pair clustering and CDR diversity. Finally, computational developability screens that analyze the amino acid sequence, structure, and combinatorial methods such as QSPR or machine learning are performed to select the most promising hits.

Several bioinformatic techniques can aid in prioritizing and selecting hits for *in vitro* confirmation of antigen binding and lead identification (Figure 3). A common strategy involves clustering hits into high-, medium-, and low-binding bins based on the initial estimates, analyzing each bin for heavy- and light-chain germline diversity, and then examining CDR diversity to select multiple representatives from each germline pair in each bin for experimental testing. Alternatively, hits can be binned based on the germline pair and CDR diversity, with selections made according to their estimated antigen binding. At this stage of hit selection, developability aspects can also be considered using computational tools introduced in the previous section. In a basic application, heavy- (HC) and light-chain (LC) sequences of hits can be scored based on the presence of potential chemical degradation motifs, aggregation-prone regions (APRs), and T-cell immune epitopes present in or overlapping with the CDRs of the heavy and light chains. The scoring schemes can be further optimized by assigning different weights based on which CDRs contain these motifs and whether they are in the Vernier zones or middle of the CDRs.

Structure-based approaches require accurate three-dimensional antibody fold information, typically generated via homology modeling. This process includes 1) identifying a high-identity structural template for framework (FW) regions, 2) loop modeling of LCDR1-3 and HCDR1-2 using canonical loop conformations, 3) HCDR3 loop modeling and optimization of the orientation of heavy-chain variable region (VH) and light-chain variable region (VL), and 4) sidechain packing and refinement. The key challenges involve obtaining high-resolution templates with optimal VH-VL orientations and accurately modeling loops, particularly the HCDR3 loop. Recent progress in Fv structure modeling has led to advanced tools, such as RosettaAntibody (Weitzner et al., 2017; Adolf-Bryfogle et al., 2018; Schoeder et al., 2021), AbPredict2 (Lapidoth et al., 2018), ABodyBuilder (Leem et al., 2016), LYRA (Klausen et al., 2015), MoFvAb (Bujotzek et al., 2015), and Kotai Antibody Builder (Yamashita et al., 2014), which demonstrate high performance in the AMA-II benchmark test. Commercial packages like Molecular Operating Environment (MOE) and BioLuminate are popular for high-throughput full-length Fv structure modeling. A detailed discussion of recent advancements in Fv structure modeling tools can be found in focused reviews (Fernández-Quintero et al., 2023). Additionally, tools like FREAD, H3LoopPred, SPHINX, MODELER, PLOP, SCWRL, BetaSCPWeb, Chothia canonical assignment, and SCALOP have significantly contributed to full-length Fv region three-dimensional structure modeling. Tools such as TopModel efficiently examine the structure for cis-amide bonds, D-amino acids, and steric clashes, allowing for rapid evaluation of model quality and accuracy prior to conducting further analysis (Norman et al., 2019; Wilman et al., 2022; Fernández-Quintero et al., 2023). The generated three-dimensional structural models of all or a subset of hits can then be analyzed regarding their physicochemical descriptors, such as pI, charge, dipole moment, and solvent-exposed hydrophobic and ionic patches. These physicochemical properties have been demonstrated to potentially influence the chemical, conformational, colloidal, and physical stabilities of antibodies, and consequently their developability. In subsequent studies, a few of the best hits are rigorously tested in the laboratory for biological function, cross-reactivity across species, non-specific binding, and pharmacological indicators, such as serum stability. This process results in the identification of one or more lead candidates.

### 3.5 Virtual screening and docking as potential alternatives to *in vitro* hit selection and lead identification

Identification of potential binders through immunization campaigns can be accomplished using bioinformatics tools for paratope and epitope prediction, followed by rapid virtual screening, as outlined in Part 2 of the *in silico* roadmap, we call DAbI (Figure 2). This approach involves three-dimensional structure modeling of a diverse antibody sequence library and screening it against a given antigen by taking advantage of the shape and charge complementarity between the epitopes and paratopes. The antibody libraries to be screened can be endowed with the biophysical characteristics desired from a developability perspective as described previously.

Small-molecule drug discovery has successfully employed virtual screening to identify binders from a library of drug candidates (Gorgulla et al., 2020; Maia et al., 2020; Yan et al., 2020). Typically, millions of small-molecule drug candidates undergo structural and energetic screening processes through docking, pharmacophore-, or ligand-based approaches. Modern techniques involving computer vision, image-based, and geometric learning–based algorithms have reached advanced stages of validation and are now well established among the marketed small-molecule drugs designed using *in silico* methods (Eguida and Rognan, 2020; Gorgulla et al., 2020; Yan et al., 2020). Similarly, a curated and modeled antibody library may be treated as a potential set of drugs to be screened against a given antigen. However, directly applying these techniques may not be feasible due to the significant structural and functional differences between small-molecule drugs and large antibodies, with size (molecular weights, 500–1,000 Da versus approximately 25,000 Da for the Fv) being a primary concern even when considering only the Fv regions. Additionally, given the estimated theoretical diversity of B-cell repertoire (BCR) based on V(D)J recombination, which is about 10^13^–10^20^ unique sequences, it is crucial to consider large antibody libraries to allow screening over a highly diverse sample space of paratopes.

Hypothetically speaking, we consider an antibody library of 1 million Fv sequences and assume a screening time of 1 min per Fv for a given antigen, the total runtime would amount to approximately 695 days (close to 23 months) for screening a single antigen against the library, which consists of only a small fraction of BCR diversity. Currently existing docking methods have runtimes of several minutes per complex. On the bright side, rapid virtual screening may not necessarily require rigorous energy-based binding evaluations employed in modern docking programs. Sacrificing the accuracy afforded by pose refinement can allow for greater speed in the screening process. Consequently, novel techniques have to be developed to enable the screening of large antibody libraries by considering the key aspects of the structural and chemical complementarity of the antigen:antibody interfaces and ensuring high-throughput rapid execution. An ideal *in silico* antibody virtual screening process could narrow down the potential binding hits to the order of 10^1^–10^2^, meaning that virtual screening would enable identifying binders at least as accurately as about one in a thousand to a few thousand sequences from the library, significantly impacting the discovery pipeline.

While *in silico* virtual screening does not replicate the generation of antigen binders via experimental methods in terms of binding affinity or functional efficacy, it can allow for comprehensive screening of the antibody library to identify all possible structural matches of epitopes and paratopes. Iterative refinement of these matches can help discover antibody binders to a given antigen with a diverse set of binding affinities and therefore suitable for antagonist as well as agonist function. Novel techniques, such as image-based and graph-based deep learning algorithms, have been proposed for identifying complementary paratope/epitope interfaces. These approaches can be further accelerated through pre-identified or predicted paratope and epitope information (Gainza et al., 2020; Pittala and Bailey-Kellogg, 2020; Akbar et al., 2021; Ripoll et al., 2021). Schneider et al. (2021) proposed a structure-based virtual screening method using voxel representation of the interfacing surface atom groups in their screening method called Deep Learning for AntiBodies (DLAB), adapted and extended from its small-molecule counterpart (Imrie et al., 2018). Recently proposed image fingerprinting–based approaches, with analogous applications in small molecules, show promising potential for protein interface matching and could be further expanded to predict paratope/epitope binders for hit selection (Gainza et al., 2020; Ripoll et al., 2021). More recently, a geometric deep learning method called ScanNet has been introduced to predict protein–protein and protein–antibody binding interfaces through geometric deep learning of three-dimensional structural features (Tubiana et al., 2022). Moreover, some of the paratope/epitope prediction methods involving deep learning of interfacial interactions may be extrapolated to interface screening and predicting binders.

The *in silico* virtual screening of antibodies against a given antigen can also borrow techniques such as fragment-based drug design (Sormanni et al., 2015; Sormanni et al., 2018) and pharmacophore modeling from the realm of small-molecule drug discovery. By facilitating the identification of binding sites, improving antibody–antigen docking, and enabling more accurate structure-based virtual screening, these methods can accelerate the development of novel therapeutic antibodies and enhance our ability to target a wider range of diseases and conditions.

Recent molecular docking protocols feature highly robust, energy-based scoring functions for evaluating and ranking protein–protein or protein–antibody binding partners. This offers a suitable toolkit for further optimization of hits identified through virtual screening of target antigens against an antibody library. Docking methods have demonstrated accurate prediction of protein-binding interfaces; however, speed has not been a priority for molecular docking programs. Although the current speed of implementation poses a bottleneck, rapid advancements in the field of protein–protein docking have spurred the development of new methods utilizing advanced machine learning algorithms and hybrid physics and learning-based technologies, promising faster docking methods soon. Moreover, such advancements may bridge the gap between virtual screening and docking, further accelerating *in silico* antibody screening, hit selection, and lead identification processes altogether.

Antibody–antigen docking has often been considered with paratope/epitope prediction and improving CDR modeling accuracy. SnugDock combines docking with accurate modeling prediction of the paratope (CDR loop construction), where the Rosetta Antibody Modeler operates alongside the docking protocol, iteratively improving docking and model prediction (Sircar and Gray, 2010; Jeliazkov et al., 2021). Additionally, methods employing more rigorous energy-defined constructs to evaluate multiple docking poses through the MM-GBSA (molecular mechanics—generalized Born solvent accessibility) method have shown promising outcomes (Shimba et al., 2016). Information-driven docking methods depend on a set of data to reduce the number of decoys, thus saving prediction time. Interface prediction-based methods, such as Antibody i-patch and EpiPred, focus on refining docking poses through paratope/epitope interface prediction (Krawczyk et al., 2013) By contrast, proABC adopts a more site-directed approach driven by the interface (paratope) (Olimpieri et al., 2013; Krawczyk et al., 2014). Advances in machine learning and deep learning algorithms have significantly contributed to enhancing docking prediction methods.

Other widely employed programs such as ClusPro, LightDock, ZDOCK, and HADDOCK, coupled with CDR and binding epitope information for directed/biased docking approaches, have shown promising results, with HADDOCK demonstrating notable performance improvement (Ambrosetti et al., 2020a). Pro-ABC-2, another information-driven docking approach and an updated version of Pro-ABC, utilizes deep learning convolutional neural networks (CNNs) for paratope prediction to assist in docking (Ambrosetti et al., 2020b). Such information-driven methods may also be applicable in pipelines using commercial docking techniques offered by MOE from Chemical Computing Group, PIPER from Schrodinger, and others with additional efforts.

Several other methods that employ deep learning through CNNs, recurrent neural networks (RNNs), or graph-based learning have demonstrated promise in predicting binding interfaces, consequently improving docking accuracy (Liberis et al., 2018; Deac et al., 2019; Pittala and Bailey-Kellogg, 2020; Lu et al., 2021; Myung et al., 2021; Vecchio et al., 2021; Davila et al., 2022). Additionally, research groups have been exploring the exceptional modeling performance of AlphaFold2 in docking prediction. The accelerated advancements in AI related to AlphaFold and other docking methods offer significant potential for the development of faster and more accurate docking programs in the future.

### 3.6 *In silico* affinity maturation of lead candidates

In a conventional discovery workflow, the lead candidates identified may have to be optimized for affinity, cross-reactivity, and developability. Among these, the focus is often on developability of the lead candidates. By contrast, DAbI may yield developable lead candidates already since the sequence and structural features that support good developability are already included in the library design (Part 1 of DAbI, see Figure 2). Depending on the library choice (antigen-agnostic or antigen-specific), the *in silico* generated lead candidate may have to be optimized for binding affinity and any residual physicochemical developability issues, particularly from the CDRs. For these reasons, the third part of our conceptual roadmap, DAbI (Figure 2), envisages an ability to adjust the binding affinity as per the project requirements. Depending upon the novel therapeutic concept (NTC), both enhancement (affinity maturation) and decrease (affinity de-maturation) in binding affinities may be required. However, affinity maturation may be required more often than de-maturation, particularly when the lead antibody binders have been derived from antigen-agnostic libraries. In our conceptual roadmap, both affinity maturation and de-maturation begin with a structural representation of the atomic interaction between two proteins, namely, the antigen and the antibody. The methodology’s reliability depends on accurately analyzing the interacting sites. Therefore, co-crystallized antibody–antigen complexes are typically preferred over structure-based homology models or AI predictions, which may lead to less reliable results if CDRs are not precisely modeled. The *in silico* affinity maturation relies on accurate molecular interactions for free energy or MM-GBSA–based calculations (Comeau et al., 2023; Thorsteinson et al., 2023), highlighting the importance of improving antibody–antigen complex predictions and the implicit incorporation of multiple conformational ensembles to enhance the effectiveness of *in silico* calculations and optimize library design. Despite this limitation, these methods have been already applied to predicted antibody–antigen complexes (Rangel et al., 2022), facilitating the generation of *in silico* affinity maturation libraries (Conti et al., 2022; Thorsteinson et al., 2023).

The *in silico* scanning of the individual paratope residues yields potential mutations and estimates of the corresponding free energy changes in binding to the target. The subsequent challenge involves designing a combinatorial assembly of these mutations into a library suitable for phage/yeast display. This is because the *in silico* affinity maturation often involves computationally expensive calculations that tend to be more accurate at identifying the single point mutations rather than combinations thereof (Comeau et al., 2023; Thorsteinson et al., 2023). The physical display libraries built using computational guidance can be used to pan combinatorial mutations. Therefore, this part of DAbI requires an understanding of the limitations associated with the library size and panning methodology (Tsumoto and Kuroda, 2022). It is also in consonance with the spirit of biopharmaceutical informatics which calls for taking advantage of the strengths of computation and experiments in a synergistic manner. When combined with library technologies like phage display, computational tools have proven particularly powerful in guiding the design of affinity maturation libraries (Tiller et al., 2017; Nelson et al., 2018; Wang et al., 2018; Thorsteinson et al., 2023). Incorporation of additional considerations along with the binding affinity can help narrow down the mutations for experimental testing and therefore the size of the display libraries. At this stage, the mutations that enhance specificity, humanness, and CDR germlining along with developability can be considered by incorporating relevant physicochemical properties and stability criteria (Khan et al., 2023; Svilenov et al., 2023). Consequently, the selection of lead antibody candidates with high binding affinity and favorable biophysical properties can be achieved simultaneously. In-house, we successfully improved binding affinities of the antibody drug candidates 10- to 1,000-fold in multiple proprietary projects using this strategy.

Several studies have demonstrated the computational design of functional antibodies using multiple structural models supported by statistical or machine learning models (Nimrod et al., 2018; Liu et al., 2019; Amimeur et al., 2020). Upon selecting an initial antibody scaffold, mutations to enhance complementarity with a given epitope can be designed to obtain specific antibody binders to an antigen. For example, the generative adversarial network (GAN) model was trained on over 400,000 light- and heavy-chain human antibody sequences to learn the rules of human antibody formation (Amimeur et al., 2020). The resulting model outperforms common *in silico* techniques, generating diverse libraries of novel antibodies mimicking somatically hypermutated human repertoire responses. Through transfer learning, the GAN can generate molecules with improved stability, developability, lower predicted major histocompatibility complex class II binding, and specific CDR characteristics. In-house, we could independently train the GAN on a much smaller set of approximately 31,500 paired antibody sequences belonging to the VH3-VK1 germline pair and format them as single chain variable regions (ScFvs). These sequences were selected based on their high percent humanness, low incidence of chemical liabilities in the CDRs, and high medicine-likeness. The in-house developed GAN model was then used to generate 100,000 unique antibody ScFv sequences and a small yet highly diverse subset of them was produced in the laboratory as immunoglobulin G1K (IgG1K) antibodies. The initial experimental characterization showed that most of the generated antibodies showed desirable attributes for expression, purification, thermal stability, and colloidal stabilities that compare favorably with those of trastuzumab, a biotherapeutic well known for its good developability profile (unpublished results). In summary, these *in silico* approaches enable the control of pharmaceutical properties for antibodies, potentially offering a more rapid and cost-effective screening, docking, and binding affinity maturation against a given target antigen.

### 3.7 Humanization and optimization of lead candidates

**FIGURE 4:**
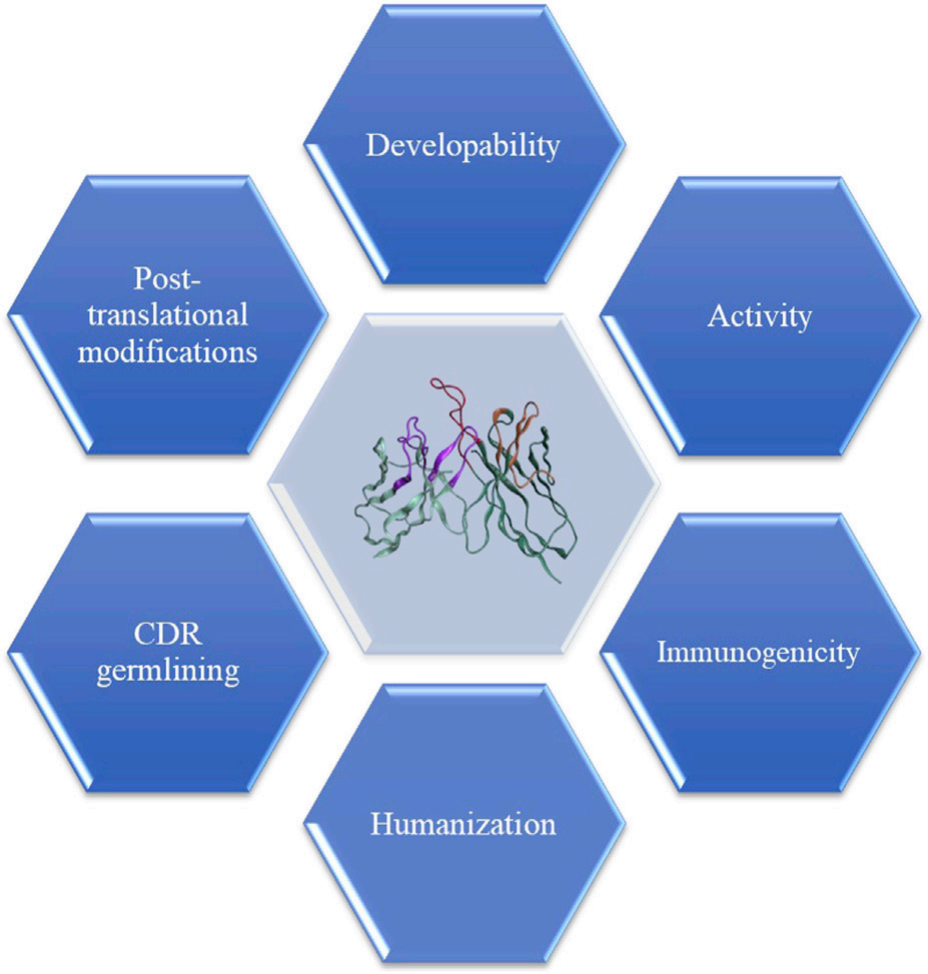
Lead humanization and optimization involve converting non-human sequences to human-like sequences while maintaining critical key attributes. *In vitro* binding affinity, which acts as surrogate for function, is the paramount criteria for accepting the mutations. Furthermore, *in silico* tools can be used to identify potential T-cell reactive epitopes, resulting in leads with lowest potential for immunogenicity and high percentage human content by germlining of the CDRs. Another aspect of optimization includes developability, which involves identifying leads with desirable biophysical properties and avoiding incidence of the post-translational modification sites such as N-linked glycosylation, unpaired cysteines, oxidation, deamidation, or aspartate isomerization, particularly in the CDRs.

During the conventional discovery workflow, lead optimization (LO) is carried out as soon as one or more lead candidates have been identified and revalidated for function. The Fv regions may require humanization if the lead molecule is from a non-human source, the removal of post-translational modification (PTM) sites, optimization of affinity, and ideally, improvement of developability (Figure 4). When all parts of DAbI are fully enabled, time and efforts required for LO may be significantly reduced, if not eliminated completely as stated earlier. However, for now, humanization and optimization of the functional lead candidates remain an integral part of biotherapeutic drug discovery. The following describes how computation can support every aspect of the LO process for therapeutic antibodies.

Humanization optimizes the amino acid sequence of non-human Fv regions, decreasing immunogenicity and anti-drug antibodies (ADAs) (Roguska et al., 1994; Townsend et al., 2015). Computational protein design methods can efficiently increase antibody humanness while maintaining structural stability (Choi et al., 2015). State-of-the-art software like MOE (ULC, 2021) enables CDR grafting and humanness optimization through *in silico* calculations (Abhinandan and Martin, 2007; Lazar et al., 2007; Gao et al., 2013; Seeliger, 2013; Olimpieri et al., 2015; Choi et al., 2017; Kuroda and Tsumoto, 2020). Bioinformatic studies have also revealed structural differences between the lambda (VL) and kappa (VK) isotypes, which must be considered during re-engineering (van der Kant et al., 2019). Structure-guided approaches can aid in enhancing the biophysical properties of a therapeutic mAb by transitioning from a problematic lambda framework (FWR) region to a more stable kappa FWR (Lehmann et al., 2015).

The humanized sequences progress to liability engineering campaigns. Pre-formulation assessments, forced degradation studies, and *in silico* evaluations are incorporated into the engineering design plan. Phage display or other screening technologies can be employed to screen a large panel of variants. *In silico* tools monitor and guide the redesign of candidates' individual liabilities (see Figure 4), and medicine-likeness can be estimated by comparing molecular characteristics with marketed antibodies (Ahmed et al., 2021).

Computational tools have successfully guided antibody optimization campaigns, improving solubility, viscosity, self-association, colloidal stability, and binding specificity (Yadav et al., 2011, 2012; Nichols et al., 2015; Kumar et al., 2018b; Shan et al., 2018; Zhang et al., 2018; Navarro and Ventura, 2019; Sakhnini et al., 2019; Bauer et al., 2020). *In silico*–guided LO campaigns have demonstrated single amino acid residue exchanges that can improve multiple chemistry, manufacturing, and control (CMC) properties, such as expression titer, yield, purity, and colloidal stability (Bauer et al., 2020). A case study enhanced antibody developability using a multi-stage approach, starting with *in silico* screening for mutations addressing liabilities while preserving thermodynamic stability, followed by production and characterization of stable candidates (Sakhnini et al., 2019). An alternative hybrid method combined computational and experimental alanine scans to identify CDR positions for mutagenesis, maintaining antigen binding and creating antibody libraries (Tiller et al., 2017). Structure-based computational designs have been effectively employed to improve the affinity and specificity of therapeutic antibodies by pinpointing the key residues in the paratope for site-directed single, double, or even triple mutations (Kiyoshi et al., 2014; Grossman et al., 2016; Kumar et al., 2018b; Chiba et al., 2020). Computational methods offer conformational stability predictions for humanization or LO (Dehouck et al., 2011; Baets et al., 2015; Folkman et al., 2016; Quan et al., 2016; Pandurangan et al., 2017; Cao et al., 2019; Leman et al., 2020), with some tools using ML on experimental data (Pandurangan et al., 2017; Cao et al., 2019). Furthermore, glycoengineering reduces aggregation propensity and enhances conformational stability of biotherapeutics (Hristodorov et al., 2013; Courtois et al., 2015).

Recommendations for amino acid substitutions help design a customized humanization and optimization strategy for the lead mAb candidate. The top lead optimized candidates (3–6) are selected for large-scale production and biophysical characterizations. These processes can be extended to multi-specific antibodies, with additional engineering for optimizing Fv or ScFv domains and identifying optimal multi-specific formats.

### 3.8 Formatting of conventional and next-generation antibodies

After optimizing Fv regions, biotherapeutic engineering proceeds with formatting Fvs into the desired antibody format, combining Fv with the chosen IgG Fc isotype. Fc engineering may be required to adjust receptor-mediated functions like antibody-dependent cell-mediated cytotoxicity (ADCC), antibody-dependent cellular phagocytosis (ADCP), complement-dependent cytotoxicity (CDC), and endosomal recycling (Mimoto et al., 2016). For next-generation biotherapeutics like bi- and multi-specific antibodies, an intermediate formatting step assesses compatibility and developability properties. Structure-based engineering supports antibody formatting, as demonstrated in a study where TGFβ1 (transforming growth factor β1) binder affinity was restored after converting from ScFv to IgG (Lord et al., 2018). Similar approaches can support formatting complex next-generation antibodies.

In the discovery process’s final step, top-performing lead variants undergo pre-formulation studies before transferring to development for cell line generation and early developability assessments (Bailly et al., 2020). The research phase concludes with the final candidate selection, after which conventional and DAbI-enabled workflows for antibody discovery are identical.

### 3.9 *In silico* assessments in early development

**FIGURE 5:**
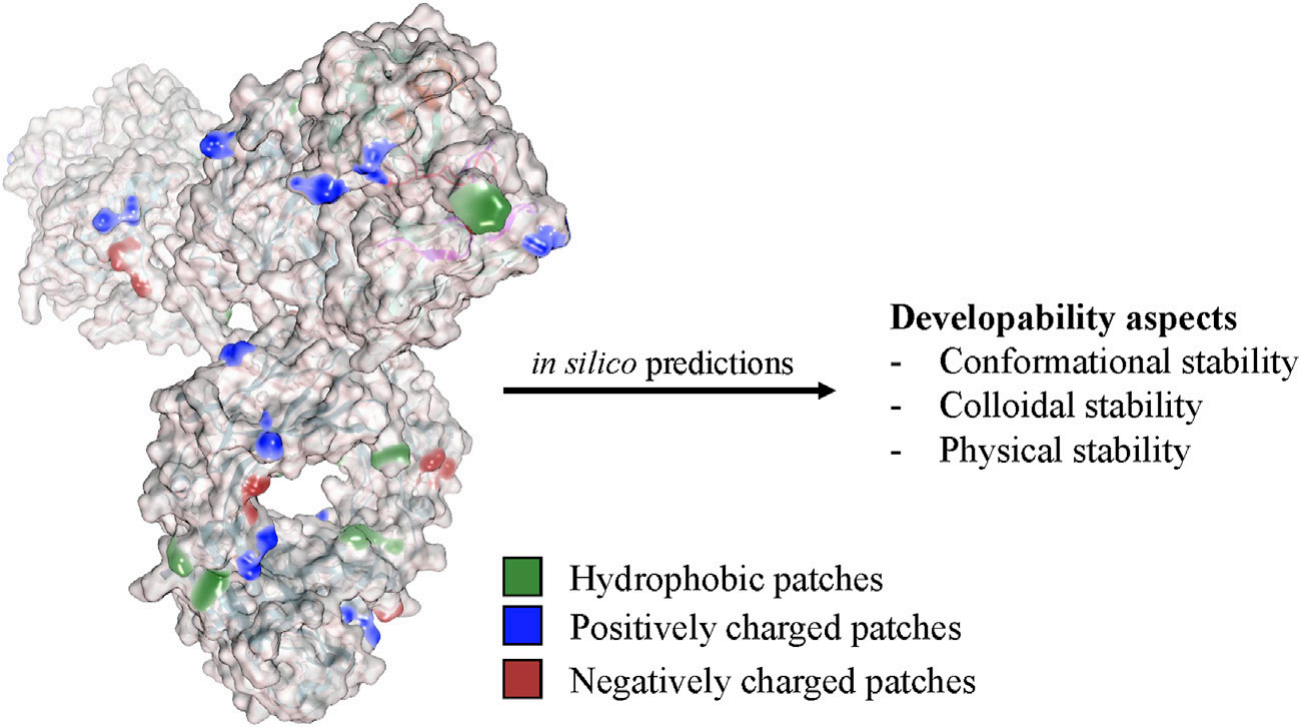
Computational approaches analyze the physicochemical properties of the antibody structure to predict various developability aspects and stability factors. These *in silico* methods evaluate factors such as aggregation propensity, conformational stability, colloidal stability, and post-translational modifications and help to select candidates with improved developability and reduced risk of immunogenicity or manufacturing challenges.

The initial stages of drug substance and drug product development are resource intensive, with full development programs justified only for the final candidate. At the time of selecting the final lead candidate, experimental data are often scarce due to material limitations. The sequence of the final lead candidate becomes locked at the start of development. This decision puts product development at a disadvantage, as real-time stability data are typically unavailable but crucial for meeting regulatory requirements concerning shelf-life, Critical Quality Attributes (CQA), and product heterogeneity. There is significant demand for early, rapid, and reliable stability predictions addressed through hybrid approaches combining *in vitro* and *in silico* techniques. Computational approaches can help estimate a molecule’s fit to specific platform processes and tailor subsequent development programs to the biologic candidate’s inherent liabilities and characteristics (Figure 5). Conversely, platform processes continuously gather data for new molecules, improving existing and developing novel bioinformatic predictions.

One platform step is the ultrafiltration/diafiltration (UF/DF), typically employed to process the antibody into the desired formulation. Recently, *in silico* models have demonstrated that protein charge can predict common UF/DF effects, such as Gibbs–Donnan and volume-exclusion phenomena (Kannan et al., 2023). After antibody formulation, certain stability aspects become most relevant for evaluating the developability of the final lead candidates using hybridized assessments.

Conformational stability is generally not an issue for conventional mAbs but can pose a significant challenge for next-generation biologics like ScFvs and multi-specific antibodies (Bailly et al., 2020). Numerous bioinformatics tools have been developed to calculate conformational stability, mostly applicable during LO for analyzing stability changes upon point mutations (Koenig et al., 2017; Pandurangan et al., 2017; Steinbrecher et al., 2017; Cao et al., 2019; Kuroda and Tsumoto, 2020; Leman et al., 2020; Harmalkar et al., 2023). Prediction accuracy heavily relies on the quality of the underlying structure or homology model, allowing comparisons between similar sequence variants.

Recent advancements in homology modeling and MD-based free energy calculations offer potential for enhancing thermal stability prediction (Kuhlman and Bradley, 2019; Berner et al., 2021; Tomar et al., 2021; Ko et al., 2022; Licari et al., 2022). Soon, these simulation approaches will extend from antibody fragments to full-length structures (Tomar et al., 2021). MD-derived predictions will improve by considering formulation aspects influencing conformational stability (Somani et al., 2021; Blanco, 2022; Saurabh et al., 2022; Shmool et al., 2022). High-throughput (HTP) screening of biologics’ thermal stabilities in platform formulations enables AI, ML, and neural networks to train computational tools to predict the thermal stabilities of diverse candidates (Gentiluomo et al., 2019a; Cao et al., 2019; Wei, 2019; Bailly et al., 2020; Harmalkar et al., 2023). The pharmaceutical industry will benefit from bioinformatic tools predicting optimal formulation composition for specific candidates or identifying the best-suited candidate for a given formulation.

Predicting colloidal stability and aggregation propensity of drug products is critical, with bioinformatics offering significant advantages in development efforts. First, real-time stability studies may take years, allowing bioinformatics to reduce development time and risk of late-stage failure. Second, stability studies require large material amounts, particularly for HCPF, increasing the cost of failures. Third, extrapolations from accelerated stability studies often inaccurately reflect molecular behavior under storage conditions. Simplified approaches using conformational stability to estimate aggregation propensity only account for non-native aggregation (Brader et al., 2015), neglecting self-association and aggregation of natively folded mAbs. Fourth, analytical techniques like HIC, dynamic light scattering (DLS), self-interaction nanoparticle spectroscopy (SINS), size exclusion chromatography (SEC), and micro-flow imaging (MFI) partially characterize colloidal instability and aggregation, often necessitating a comprehensive analytical panel (Kopp et al., 2020). Last, colloidal instability and aggregation can be triggered by various intrinsic (molecule-related) (Alam et al., 2019; Gentiluomo et al., 2019b; Lai et al., 2022) and extrinsic (process-related) factors, following complex mechanisms. Conventional methods struggle to accurately predict shelf-life, leading to resource-intensive development studies and troubleshooting efforts when the development success is at risk.

A thorough understanding of molecular behavior is essential for addressing self-association, aggregation, or particulate formation issues. Computational approaches have been developed to estimate mechanistic and kinetic characteristics for better comprehension and prediction of colloidal instability and aggregation. Mechanistic tools aid in screening and minimizing APRs during the discovery phase (Kuhn et al., 2017; Prabakaran et al., 2017, 2020; van der Kant et al., 2017; Gil-Garcia et al., 2018; Rawat et al., 2018; Bauer et al., 2020; Ebo et al., 2020; Shahfar et al., 2022), while kinetic predictors estimate aggregation rates, crucial for liquid formulation development meeting regulatory requirements for shelf life (Rawat et al., 2019; Yang et al., 2019; Santos et al., 2020). Machine Learning (ML) can train kinetic models using extensive data sets with experimental and sequence/structure information (Rawat et al., 2019; Yang et al., 2019), facilitating prediction of optimal formulation compositions (pH, salt, excipients) for minimal kinetics.

In the final development stage, creating liquid drug products with stable physical properties is vital. Manufacturing, processing, and administration of highly concentrated antibody formulations often face viscosity challenges. Viscosity is linked to surface charge and hydrophobicity of the mAb (Tomar et al., 2018; Apgar et al., 2020; Lai et al., 2021; Blanco, 2022; Han et al., 2022; Lai, 2022). Studies have shown computational ability to predict viscosity profiles at platform conditions using mAb sequence and structure (Tilegenova et al., 2019; Bauer et al., 2020; Thorsteinson et al., 2021; Han et al., 2022; Lai et al., 2022; Rosace et al., 2022). A recent deep learning approach utilized a 3D convolutional neural network to predict high-concentration viscosity of therapeutic antibodies (Rai et al., 2023). Feature attribution analysis identified key biophysical drivers of viscosity, such as the electrostatic potential surface. The predictor was successfully trained despite limited data. Early integration of viscosity predictors enables addressing viscosity issues and adjusting platform formulations and technologies before finalizing the development strategy.

## 4 Discussion and conclusion

In this review, we have presented numerous opportunities for computation to play a greater role in biotherapeutics discovery and development. However, the excitement around computation’s enhanced role should be tempered with pragmatism. Machine learning experts often lack practical experience in biotherapeutics discovery and development and *vice versa*. Thus, a strong collaboration between bench scientists and data scientists is recommended. Computational biophysics and antibody structure–function–developability relationship experts should work with machine learning and artificial intelligence experts, as well as experimentalists, to fully enable biopharmaceutical informatics. Additionally, technical limitations exist in emerging technologies like machine learning and artificial intelligence. For instance, deep learning model performance often depends on size and diversity within training data sets (Wittmund et al., 2022), posing challenges in sparse or less diverse data settings. Moreover, the lack of insights into the latent space and interpretability of AI models in terms of the underlying physicochemical rules hinders our ability to better understand the models and extend their applicability beyond the tasks they have been trained for. For example, AI-based methods have transformed protein structure prediction, but contrary to popular belief, they have not solved the protein folding problem (Chen et al., 2023), as they do not provide insights into protein folding processes, such as initial building blocks, intermediate states, energy landscapes, and pathways.

In the specific context of protein engineering, the complexity of prediction tasks is escalated by non-additive mutational interactions or epistatic effects, which can significantly alter the impact of single or multiple mutational outcomes (Reetz, 2013; Miton and Tokuriki, 2016; Cadet et al., 2022). A further layer of complication is presented by the dynamic interplay between mutated amino acids and the subsequent establishment of intramolecular interaction networks, which can alter the protein function (Acevedo-Rocha et al., 2021). The situation is exacerbated by the limitations of tools such as AlphaFold2 or ProteinMPNN, which may struggle to predict how individual amino acid changes affect protein structure due to their heavy reliance on evolutionary perspectives and variant sequences (Eisenstein, 2021; Dauparas et al., 2022). Deep learning methods offer a way to investigate protein attributes, such as stability, solubility, aggregation, and binding affinity. However, these methods operate within the confines of the training data. Although this does not eliminate the possibility of identifying beneficial protein variations within these parameters, it may fail to recognize or accurately predict variants exhibiting fitness values outside the learned range. This means that while beneficial variants can be identified, the optimal variant, particularly if it is an epistatic variant, might be overlooked. Against this backdrop, the use of deep learning models in conjunction with conventional neural network architectures is being explored as a solution for these challenges. By representing numerical quantities as individual neurons without non-linearity, these models can learn to perform systematic numerical computation, enabling them to handle data that lie outside the range used during training (Trask et al., 2018). The adaptability of these models across various task domains augments their potential to tackle challenges encountered in antibody therapeutics. Importantly, the ability to harness epistatic effects and predict mutational outcomes could significantly enhance the design of therapeutic antibodies. Moreover, other studies have indicated the potency of a Machine learning (ML) approach focused exclusively on sequences in accurately predicting epistatic phenomena (Cadet et al., 2018). Unlike most ML and deep learning methodologies that predominantly capture low-order non-linear interactions and predict the additive effects of mutations, this innovative strategy comprehensively encapsulates both low- and high-order non-linear interactions. By utilizing ML in tandem with digital signal processing such as Fourier transform, case studies have demonstrated a significant improvement in the resistance of proteins to unfavorable unfolding and aggregation. Crucially, this method unveils the correlation between epistatic mutational interactions and protein resilience, offering unique, predictive insights beyond those provided by conventional machine learning or deep learning approaches (Li et al., 2021). This approach has considerably enhanced precision, reduced overfitting, and surpassed conventional methods without increasing complexity (Medina-Ortiz et al., 2022). Understanding the rules underlying these interactions could contribute to a more efficient model design and a more predictive performance, thereby bolstering the success of deep learning in the realm of biopharmaceutical informatics.

In conclusion, this review article aims to broaden our strategic perspective on biopharmaceutical informatics. Initially, we emphasized the syncretic use of computation and experimentation for the drug product development of antibody-based biotherapeutics (Kumar et al., 2015; Kumar et al., 2018a). Subsequently, Khetan et al. (2022) demonstrated its feasibility by spelling out different methods and published studies already available in the public domain to support our vision. Here, we propose a more generalized vision of biopharmaceutical informatics by including DAbI and digital transformation. It is widely agreed that digital transformation is essential for modernizing the biopharmaceutical industry’s work processes, leading to more judicious use of resources and reduced costs in biotherapeutics discovery and development. Recent advancements in AI and ML, along with the availability of large-scale antibody sequencing data in the public domain, have fueled excitement for DAbI. When fully embraced by the biopharmaceutical industry, DAbI will revolutionize the way biotherapeutic drugs are discovered and developed. Current drug discovery processes and workflows are dominated by experimental trials and errors, with computation playing an assistive role at the best. DAbI can support the start of projects even before the availability of antigen material for *in vitro* experimental studies. This is particularly attractive when the antigens involved are difficult to express and purify. DAbI can also accelerate discovery projects by pre-paying for developability and therefore save on resources and time required to fix these issues at the later stages. These two features may eventually lead to situations where computation plays an equal, if not greater, role alongside experimentation in supporting biotherapeutics discovery and development projects. Therefore, our vision of biopharmaceutical informatics points to an exciting future where we can better serve patients by addressing unmet medical needs through more successful, faster, and affordable discovery and development of biotherapeutics. Additionally, the discovery and development of antibody-based biotherapeutics are rapidly becoming industrialized, with several aspects becoming more uniform (e.g., discovery processes and drug formulations), while multiple options are being explored for others, such as molecular formats, routes of administration, and dosing options (Martin et al., 2023). Biopharmaceutical informatics contributes toward accelerating this industrialization and helping to improve human health.

## Figures and Tables

**Table:** 

**Ab**	Antibody
**ADA**	Anti-drug antibodies
**ADCC**	Antibody-dependent cell-mediated cytotoxicity
**ADCP**	Antibody-dependent cellular phagocytosis
**Ag**	Antigen
**AI**	Artificial intelligence
**Ala**	Alanine
**APR**	Aggregation-prone region
**BCR**	B-cell repertoire
**CDC**	Complement-dependent cytotoxicity
**CDR**	Complementarity determining region
**CMC**	Chemistry, manufacturing, and control
**CNN**	Convolutional neural networks
**CQA**	Clinical Quality Attributes
**DAbI**	Discovery of antibodies *in silico*
**DENIS**	DEvelopability Navigator *In Silico*
**DLAB**	Deep Learning for AntiBodies
**DLS**	Dynamic light scattering
**Fab**	Fragment antigen binding
**FAIR**	Findable, accessible, interoperable, and reusable
**FDA**	Food and Drug Administration
**Fv**	Fragment variable
**FW**	Framework
**GAN**	Generalized adversarial network
**HC**	Heavy chain
**HCDR1-3**	Heavy-chain complementarity determining regions 1–3
**HCPF**	High-concentration protein formulations
**HIC**	Hydrophobic interaction chromatography
**HTTP**	High-throughput
**IgG**	Immunoglobulin G
**LC**	Light chain
**LCDR1-3**	Light-chain complementarity determining regions 1–3
**LO**	Lead optimization
**mAb**	Monoclonal antibody
**MaSIF**	Molecular surface interaction fingerprints
**MD**	Molecular dynamics
**MFI**	Micro-flow imaging
**MHC**	Major histocompatibility complex
**ML**	Machine learning
**MM-GBSA**	Molecular mechanics—generalized Born solvent accessibility
**MOE**	Molecular Operating Environment
**NBE**	New biologic entity
**NTC**	Novel therapeutic concept
**pI**	Isoelectric point
**PTM**	Post-translational modification
**QSAR**	Quantitative structure–activity relationship
**QSPR**	Quantitative structure–property relationship
**R&D**	Research and development
**RNN**	Recurrent neural networks
**RTP**	Research target profile
**ScFv**	Single-chain fragment variable
**SCM**	Spatial charge map
**SEC**	Size exclusion chromatography
**SINS**	Self-interaction nanoparticle spectroscopy
**TGFβ1**	Transforming growth factor β1
**UF/DF**	Ultrafiltration/diafiltration
**VH**	Heavy-chain variable region
**VK**	Light-chain variable region (kappa isotype)
**VL**	Light-chain variable region (lambda isotype)
